# Case of Fracture of the Skull

**Published:** 1857-03

**Authors:** Darwin Colvin

**Affiliations:** Clyde, N. Y.


ART. III.— *Case of Fracture of the Skull.* By DARWIN COLVIN, M. D., Clyde, N. Y.

*Editor Journal:* Permit me to send you a rude history of a case of fracture of the skull, extending across its base, which has recently come under my care, and which to me was, in many respects, interesting:

J. B., aged 37, while leading a horse by the halter, on the morning of the 26th of January, received a kick upon the external angular process of the left superciliary ridge, and in falling, the vertex struck (as those present say) with great force upon the iron rail of a temporary railroad track, which he was crossing.

The scene of the accident being six miles from me, some time had elapsed before I saw him. Upon arriving, we (Dr. M. P. Colvin, in consultation,) ascertained that there was a compound fracture of the frontal bone, with no depression. Profuse hæmorrhage from the nose and both ears; sensibility incomplete; surface cool; pupils uniform and slightly dilated; pulse 56, and soft; had vomited once; breathing quiet.

Suspecting a fracture of the base, the treatment was expectant; closed the wound.

6, P. M. No particular change since morning; skin a little warmer.

Shaved the scalp and directed ice-cold water to be constantly applied to it; mustard poultices to the lower extremities; $\frac{1}{4}$ gr. morphia, *pro re nata.*

27th, 9, A. M. Pulse 68, full and soft; tongue dry; skin warm; hæmorrhage from the nostrils and one ear ceased at 2 o'clock this morning, but continues from the other; pupils natural; countenance yellow; recognizes his family and friends.

Directed 10 grs. calomel to be given, and followed in three hours with one ounce of sulph. magnes., after the operation of which continue treatment as before.

6, P. M. Cathartic has operated much to the patient's relief. Secretion of urine copious; skin cool; pulse 60; tongue moist; icterus increases; complains of his back; says he is hungry; hæmorrhage from the ear has ceased.

28th and 29th. No special change, but is evidently suffering from the effects of a serious lesion of the skull; still complains of his back.

30th, 6, A. M. Rested well until 4 this morning, when he began to exhibit symptoms of delirium; wants to go home; complains of his head and back; pupils unchanged; pulse 56, and still soft; skin warm and intensely yellow; no general fever.

Take 1 oz. sulph. magnes., after which continue treatment, with the addition of $\frac{1}{4}$ gr. tart. ant. et potass., once an hour if skin should become hot, and 2 grs. cal. once in three hours.

6, P. M. Cathartic has operated; patient more quiet; skin, pulse, &c., as in the morning.

31st, 9, A. M. Has rested better, but at times is delirious. Symptoms generally as yesterday.

Continue treatment, with a blister, 3 × 6, to the arm.

Feb. 1st and 2d. No apparent change in symptoms. No alteration in treatment. Blister to the other arm. Complains of the first blister and of his back.

3d, 9, A. M. Symptoms materially worse this morning; is constantly getting up and down and *walking about the lower rooms* of the house; will generally go to bed when asked, but immediately gets up. Skin cool; pulse 58, and soft; pupils slightly contracted; puts his hand to his head and says, "Dr., there is the trouble;" says he is very hungry, which by the way has been an unceasing complaint.

Increased the morphia.

4th, 9, A. M. Has been more quiet until within a short time, when he again began to walk the different rooms. Does not recognize his family; no change in condition of skin; pulse 82, and soft.

No change in treatment.

6, P. M. Evidently failing; *this afternoon* called his child to him and kissed it, and talked for a short time to his employer; vision nearly lost; I think he will not live through the night.

5th, 9, A. M. Is still alive, but unconscious of what is transpiring about him; head hot for the *first time*; pupils contracted; skin generally cool; pulse irregular and soft; bowels have moved; secretion of urine copious.

No particular treatment.

6th, 9, A. M. Patient died at 4, this morning. I ascertained that he had been quiet, (not comatose,) up to 3½, A. M., when he had what the nurses supposed to be a chill, which lasted half an hour, and immediately expired.

Autopsy twelve hours after death.

Upon dissecting off the scalp, a large coagulum of blood was found beneath it, and over the vertex, at the point of skull which came in contact with the rail at the time of falling.

Pus was discharging from the fracture.

Upon removing the calvarium and brain, the fracture was observed to extend backwar to about the middle of the temporal bone. From about one inch from the beginning of the fracture, another was seen traversing the base of the skull, passing through the sella turcica upon the opposite side; thence backward, crossing the petrous portion of the temporal bone, and terminating in the meatus auditorius internus; about half an ounce of pus was found beneath the brain.

Upon looking over these notes I find that I have omitted to mention one symptom which seemed to confirm my belief that there was in this case a fracture at the base, to wit: an inability to elevate the lid of the affected side; at first I attributed it to the fact that it was much thickened from extravasation, but after that had disappeared, there was the same want of power, which continued up to his death. No doubt it was owing to a lesion of the third pair of nerves, as the fracture invaded the foramen through which it passes. I have no doubt but that the fracture was increased by the fall.

If there is anything of surgical interest in this case, will you oblige me (after having smoothed the rough surfaces) by giving it a place in your Journal, at some future time; if not, let it share the fate of scores of others of like character, which, no doubt, you are daily receiving.

